# Human-stool-associated tusavirus (*Parvoviridae*) in domestic goats and sheep

**DOI:** 10.1007/s00705-022-05424-8

**Published:** 2022-03-31

**Authors:** Gábor Reuter, Péter Pankovics, Zoltán László, Gábor Gáspár, Alvin Hui, Eric Delwart, Ákos Boros

**Affiliations:** 1 Department of Medical Microbiology and Immunology, Medical School, University of Pécs, Szigeti út 12., Pécs, 7624 Hungary; 2 Vitalant Research Institute, San Francisco, CA USA; 3 University of California, San Francisco, CA USA

## Abstract

In this study, genetic counterparts of the human-stool-associated tusavirus (*s*ubfamily *Parvovirinae,* family *Parvoviridae*) with >97% and 95-100% amino acid sequence identity in the parvoviral NS1 and VP1 protein were identified in faecal specimens from domestic goats (*Capra hircus*) and sheep (*Ovis aries*) in Hungary. Eleven (17.8%) of the 62 faecal specimens from goats and 12 (25.5%) of the 47 from sheep both from less than 12 months old animals were positive for tusavirus DNA by PCR, while none of the specimens collected from cattle and swine were positive. Thus, it cannot be ruled out that tusavirus infection in humans is of zoonotic origin.

**Supplementary Information:**

The online version contains supplementary material available at 10.1007/s00705-022-05424-8.

Parvoviruses are genetically diverse non-enveloped viruses with an approximately 4- to 6-kb-long single-stranded DNA genome that infect a wide diversity of animals ranging insects to humans [1]. The family *Parvoviridae* contains three subfamilies: *Densovirinae*, whose members infect invertebrates, and *Parvovirinae* and *Hamaparvovirinae*, whose members infect vertebrate hosts [1, 2]. These subfamilies currently consist of 11, 10, and 5 genera, respectively [2].

Tusavirus is among the newest parvoviruses discovered in human samples and belongs to the genus *Protoparvovirus, s*ubfamily *Parvovirinae*. Tusavirus (Tunisian stool-associated parvovirus) was first identified in a single faecal specimen from an 18-month-old Tunisian child with unexplained diarrhoea in Africa [3]. Since then, there has been little research on human tusavirus. Tusavirus DNA was subsequently detected in two faecal samples from Finnish adults in their twenties with gastroenteritis [4]. The seroprevalence in the Finnish population is low, 0.44% (1/228) in children [5], 0% (0/180) in adults [5, 6], and 0.8% (1/124) in transplant patients [7]. Furthermore, in a more extensive study, tusavirus IgG was not detected in any human serum samples (n = 840) collected from five countries (Finland, USA, Iran, Iraq, and Kenya) on four continents [8]. Thus, tusavirus infections in humans appear to be rare, and the potential non-human origin and diversity of tusavirus has not been investigated.

In this study, a parvovirus genome genetically closely related to human-stool-associated tusavirus was identified in a high percentage of faecal samples from goats and sheep in Hungary. Animals could therefore be a potential zoonotic source of tusavirus infections of humans.

A specimen pool containing three faecal samples (KT-G4, KT-G5, and KT-FI-3) from goats (*Capra hircus*) with gastroenteritis were analyzed by viral metagenomics. Briefly, 200 μl of the PBS-diluted specimen was passed through a 0.45-μm sterile filter (Millipore) and centrifuged at 6,000 × *g* for 5 min. Then, the filtrate was treated with a mixture of DNases and RNases to digest unprotected nucleic acids at 37°C for 2 hours. Enriched viral nucleic acids (RNA and DNA) were then extracted and amplified using a Nextera XT DNA Library Preparation Kit (Illumina). The library was sequenced on an Illumina MiSeq platform. The metagenomic reads were trimmed, assembled *de novo*, and analyzed using an in-house bioinformatics pipeline [9]. Singlets and assembled contigs greater than 250 bp were compared to the GenBank protein database using BLASTx, yielding 121,343 unique sequences.

A total of 233 raw reads and contigs from the goat sample pool showed similarity to parvovirus (family *Parvoviridae*) sequences, with the highest >82% sequence identity to human tusavirus (KJ495710). Tusavirus DNA was then identified by PCR in two of the three samples (KT-G4 and KT-G5). The nearly complete, 4,316-nucleotide (nt)-long genome sequence (goat tusavirus/KT-G5/2020/HUN, GenBank accession no. OL692339) was obtained and found to contain a partial 3’ untranslated region (102 nt), complete NS1 replicase (625 amino acid [aa]) and viral protein 1 (VP1, 715 aa) genes, and a partial 5’ untranslated region (98 nt). All of the characteristic amino acid sequence motifs present in human tusavirus [3] were also identifiable in goat tusavirus, including the same start codons in NS1 and VP1. The NS1 encodes the viral helicase, including the conserved ATP- or GTP-binding Walker A loop (GxxxxGKT/S; _396_**G**PATT**GKS**_403_), Walker B (xxxxEE; _436_IIWV**EE**_441_), Walker B’ (KxxxxGxxxxxxxK; _453_**K**AICS**G**QTIRIDQ**K**_466_), and Walker C (PIxIXXN; _477_**P**VIMTT**N**_483_) aa motifs. In addition, the NS1 protein contains of two conserved replication initiator (endonuclease) motifs: xxHuHxxxx (EF_129_**H**V**H**_131_VLLW) and YxxxK (_212_**Y**FLR**K**_216_). The N-terminal GPGN calcium-binding loop (_20_**GPGN**_23_) and the phospholipase A_2_ (PLA_2_) DxxAxxHDxxY catalytic residues (_35_**D**AA**A**RR**HD**FA**Y**_45_), which are widely present in the VP1 unique part (VP1up) of many parvoviruses [10], are identifiable in the VP1 protein of goat tusavirus/KT-G5/2020/HUN. The goat tusavirus/KT-G5/2020/HUN NS1 protein shares 84% nt and 96% aa (97% aa positives) sequence identity with corresponding NS1 genome region and protein, respectively, and the VP1 shares 82% nt and 89% aa (95% aa positives) sequence identity with the corresponding VPI genome region and protein, respectively, of human tusavirus (KJ495710).

To determine the prevalence of tusavirus in domestic animals, a total of 234 faecal samples from goats (N = 62), sheep (N = 47), cattle (N = 95), and swine (N = 30) of different age groups, with or without diarrhoea, were collected from 20 farms in Hungary (Supplementary Table S1) and tested by PCR and sequencing using screening primers (tusavirus-VP1-F: 5’-CACCAGCTGCTAGACCACGA-3’; tusavirus-VP1-R: 5’-CGCGAGCACCTCCTCCTGAA-3’) designed to recognize the N-terminal part of the VP1 region of both human (KJ495710) and goat tusavirus KT-G5 (OL692339) sequences. Eleven (17.8%) of the 62 specimens from goats (in two farms) and 12 (25.5%) of the 47 specimens from sheep (in one farm) both from less than 12 months old animals were positive for tusavirus by PCR (Table 1). Of the 11 goats with tusavirus PCR-positive specimens five had diarrhoea and six were asymptomatic at the time of sampling. On the other hand, all 12 tusavirus-PCR-positive specimens were collected from asymptomatic sheep (Supplementary Table S1). None of the specimens from cattle or swine were positive for tusavirus (Table 1, Supplementary Table S1).

**Table 1:** Detection of tusavirus by PCR and its distribution in faecal specimens collected from domestic animals from 20 farms in Hungary, including goats, sheep, cattle, and swine

Host species	Farm location	Farm ID	Collection date (mm/dd/yy)	No. of positive faecal samples/total by age group			No. of faecal samples PCR-positive for tusavirus/total (%) by farm
				<2 month	2-12 month	>12 month	
Goat	Aranyosgadány	AGR	04/23/2020	NA	0/8	0/8	0/16
	Győrszentiván	KT	05/11/2020	0/9	10/10	0/10	10/29 (34.5%)
	Nagyhegy	NH	05/11/2020	NA	NA	0/5	0/5
	Rudabánya	K	06/25/2008	NA	1/12	NA	1/12 (8.3%)
Total				0/9	11/30 (36.7%)	0/23	**11/62 (17.8%)**
Sheep	Hajdúszoboszló	HBSZ	03/05/2020	0/5	0/8	0/6	0/19
	Tárnok	TB	03/16/2009 04/02/2010	7/8 5/8	NA	NA	12/16 (75.0%)
	Békéscsaba	ANI	09/06/2009	NA	NA	0/12	0/12
Total				12/21 (57.1%)	0/8	0/18	**12/47 (25.5%)**
Cattle	Hajdúböszörmény	HB	03/05/2020	0/6	0/13	0/2	0/21
	Nyíregyháza	NyH	03/06/2020	0/7	NA	0/4	0/11
	Derecske	DR	03/05/2020	NA	0/4	0/11	0/15
	Tiszavasvári	TiV	03/05/2020	0/6	0/9	0/1	0/16
	Bonyhád	BH	11/04/2019	0/14	0/2	NA	0/16
	Tevel	TV	11/04/2019	0/13	0/3	NA	0/16
Total				0/46	0/31	0/18	**0/95**
Swine	Egyházasfalu	EF	08/17/2016	NA	0/10	NA	0/10
	Tázlár	TM	01/2013	0/3	NA	NA	0/3
	Nagyszokoly	NSzK	01/23/2013	NA	0/1	NA	0/1
	Kevermes	KEV	08/05/2013	NA	0/3	NA	0/3
	Zana	Zs	04/14/2013	NA	0/3	NA	0/3
	Balmazújváros	BUV	04/24/2013	0/2	NA	NA	0/2
	Székelyszabar	SZ	01/2013	0/4	NA	0/4	0/8
Total				0/9	0/17	0/4	**0/30**

Detailed data related to each animal are available in Supplementary Table S1

NA no sample available

The nt sequence identity values were 100% and 97% among goat tusaviruses identified within and between farms, respectively. The nearly complete nucleotide sequence of the VP1 coding region of sheep tusaviruses (TB1, TB5, TB7, TB9, TB10, and TB14) was determined. The nt sequence identity in VP1 was 100% among sheep tusavirus TB1, TB5, and TB7 collected in 2009, but the otherwise identical sequences from TB9, TB10, and TB14 (collected in 2010 from the same farm, Tárnok) showed a 9% nt (and 1% aa) difference compared to the 2009 sequences. The sheep tusavirus VP1 sequences were found to have 91-97% nt and 99-100% aa sequence identity to the corresponding VPI gene and protein sequences, respectively, of human-stool-associated tusavirus (KJ495710).

Phylogenetic analysis based on VP1 aa sequences showed that tusaviruses (OL692339-OL692348) from goat and sheep are very closely related to each other and to the human tusavirus (Fig. 1).

**Fig. 1:**
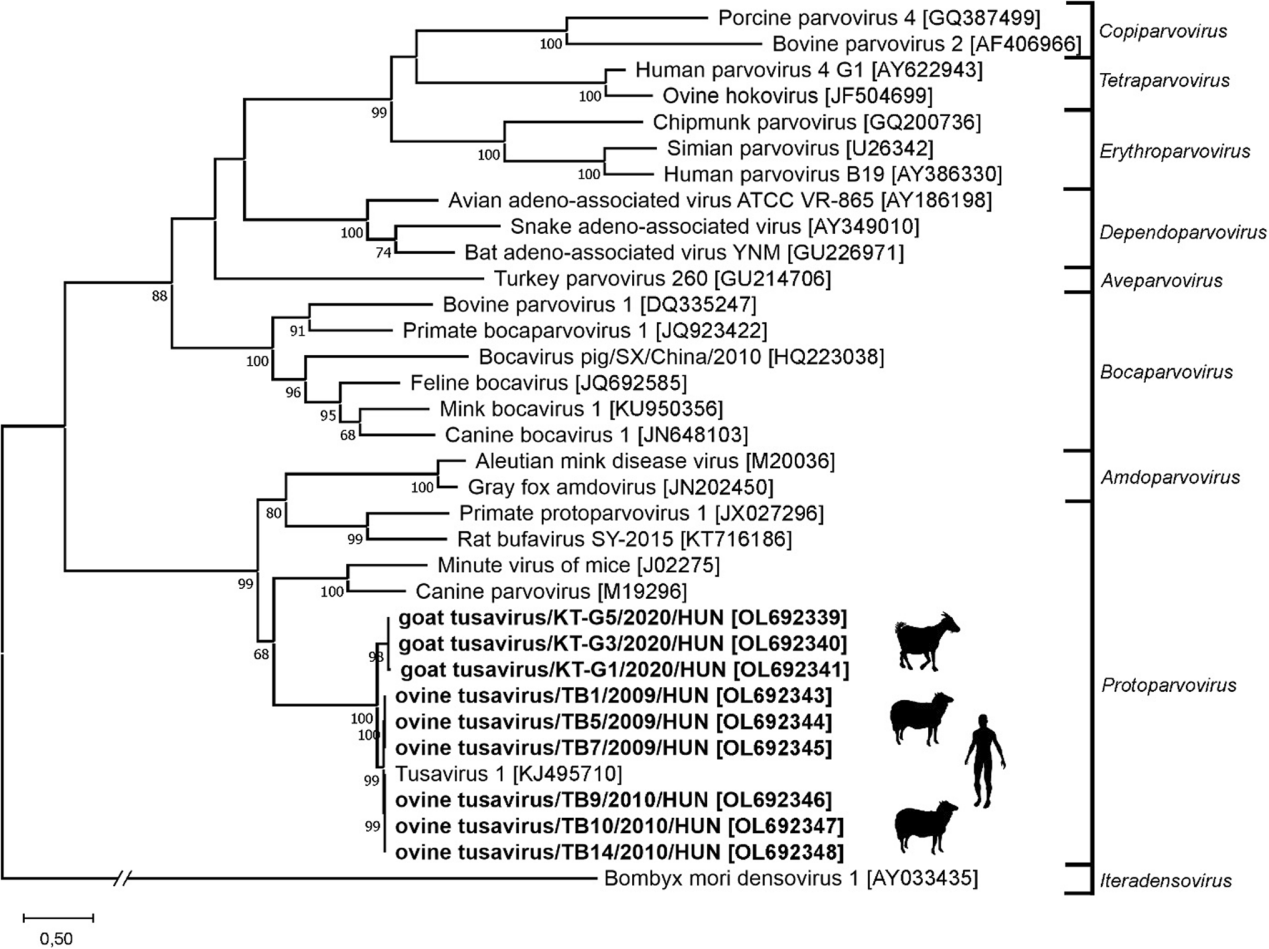
A phylogenetic tree based on a MUSCLE alignment [11] of the VP1 capsid amino acid sequences of representative members of the subfamily *Parvovirinae* and the tusavirus strains from this study (in bold). The tree was generated from the alignment using the maximum-likelihood method and the general reverse transcriptase **????** + Freq. model with 1000 bootstrap replicates, which was selected based on the results of a best-model search in MEGA ver. 10.2.1 [12]. Bootstrap values less than 50 were omitted from the tree. The host species of the studied viruses (from goat and ovine) and the known tusavirus from humans are indicated by silhouettes.

Underlining the importance of a One Health approach, the discovery in goats and sheep of very close relatives of a recently characterized parvovirus found in human faeces [3] indicates its potential zoonotic origin. The tusavirus sequence from goats (OL692339) represents the second coding-complete genome sequence of a tusavirus, with human-stool-associated tusavirus (KJ495710) being the first. We cannot exclude the possibility of other domestic and/or wild animals also being reservoirs and hosts for tusavirus.

In the light of the finding of novel tusavirus genomes in animals, the prevalence of tusavirus in human faeces might have been underestimated, since the nested-PCR primers used by Phan et al. [3] and both primers and the probe used for molecular detection by Väisänen et al. [7] and Mohanraj et al. [4] contained 2-6 nucleotide mismatches relative to the animal tusaviruses. Antigenic diversity may have similarly affected the results of human seroepidemiological prevalence studies.

Tusavirus was found in a high percentage of faecal samples from goats and sheep in Hungary. Tusaviruses from humans, goats, and sheep have a high degree of genetic and possibly antigenic similarity. Based on the capsid sequence and structure comparison, tusavirus is a “hybrid virus” with molecular characteristics lying between those of primate and non-primate protoparvoviruses [13]. Tusavirus was also found to display affinity to a wider set of glycans than human cutavirus [4] based on its sialic acid cell receptor binding pattern [13]. These results indicate that tusavirus has an extended host range and raises the possibility that human tusavirus infections could be zoonotic in origin.

Detection of tusavirus in domestic animals is an important step forward in tusavirus research. Further studies will be required to investigate the full spectrum of animal reservoirs, genetic and antigenic diversity, and pathogenesis of tusaviruses – from both the medical and veterinary points of view – in human as well as in domestic and wild animals.

## Supplementary Information

Below is the link to the electronic supplementary material.

Supplementary file1 (DOCX 46 KB)
