# Supplementary material for: Human-stool-associated tusavirus (Parvoviridae) in domestic goats and sheep
Source: Arch Virol. 2022 Mar 31;167(5):1307–10. doi: 10.1007/s00705-022-05424-8 (PMC9038789; doi:10.1007/s00705-022-05424-8)
Supplement: Supplementary file 1 — Supplementary file1 (DOCX 46 KB) [file 705_2022_5424_MOESM1_ESM.docx]

**Table S1**: Detailed background information of faecal samples used for the epidemiological investigations of tusaviruses as well as the results of tusavirus PCR screening (neg.: negative, **Pos.**: positive). ID: identification marks. The age group I: < 2 month-old animals, Age group II. 2-12-month-old animals, Age group III: >12 month-old animals. Samples with available tusavirus genome sequences (nearly complete genome or complete/partial VP1) deposited in the GenBank database are marked with *.

| **Farm location (ID)** | **Sample ID*** | **Species** | **Age** | **Age group** | **Health status** | **Collection date** | **Tusavirus PCR** |
| --- | --- | --- | --- | --- | --- | --- | --- |
| Hajdúszoboszló (HBSZ) | HBSZ-B-GI-1 | ovine | 3 months | II | asymptomatic | 03/05/2020 | neg. |
| Hajdúszoboszló (HBSZ) | HBSZ-B-GI-2 | ovine | 3 months | II | asymptomatic | 03/05/2020 | neg. |
| Hajdúszoboszló (HBSZ) | HBSZ-B-GI-3 | ovine | 3 months | II | asymptomatic | 03/05/2020 | neg. |
| Hajdúszoboszló (HBSZ) | HBSZ-B-GI-4 | ovine | 3 months | II | asymptomatic | 03/05/2020 | neg. |
| Hajdúszoboszló (HBSZ) | HBSZ-B-GI-5 | ovine | 3 months | II | asymptomatic | 03/05/2020 | neg. |
| Hajdúszoboszló (HBSZ) | HBSZ-B-GII-1 | ovine | >2 months | I | asymptomatic | 03/05/2020 | neg. |
| Hajdúszoboszló (HBSZ) | HBSZ-B-GII-2 | ovine | >2 months | I | asymptomatic | 03/05/2020 | neg. |
| Hajdúszoboszló (HBSZ) | HBSZ-B-GII-3 | ovine | >2 months | I | asymptomatic | 03/05/2020 | neg. |
| Hajdúszoboszló (HBSZ) | HBSZ-B-GII-4 | ovine | >2 months | I | asymptomatic | 03/05/2020 | neg. |
| Hajdúszoboszló (HBSZ) | HBSZ-B-GII-5 | ovine | >2 months | I | asymptomatic | 03/05/2020 | neg. |
| Hajdúszoboszló (HBSZ) | HBSZ-B-GIII-1 | ovine | 7-8 months | II | asymptomatic | 03/05/2020 | neg. |
| Hajdúszoboszló (HBSZ) | HBSZ-B-GIII-2 | ovine | 7-8 months | II | asymptomatic | 03/05/2020 | neg. |
| Hajdúszoboszló (HBSZ) | HBSZ-B-GIII-3 | ovine | 7-8 months | II | asymptomatic | 03/05/2020 | neg. |
| Hajdúszoboszló (HBSZ) | HBSZ-B-GIV-1 | ovine | 2-6 years | III | asymptomatic | 03/05/2020 | neg. |
| Hajdúszoboszló (HBSZ) | HBSZ-B-GIV-2 | ovine | 2-6 years | III | asymptomatic | 03/05/2020 | neg. |
| Hajdúszoboszló (HBSZ) | HBSZ-B-GV-1 | ovine | 2-6 years | III | asymptomatic | 03/05/2020 | neg. |
| Hajdúszoboszló (HBSZ) | HBSZ-B-GV-2 | ovine | 2-6 years | III | asymptomatic | 03/05/2020 | neg. |
| Hajdúszoboszló (HBSZ) | HBSZ-K-1 | ovine | 2.5 years | III | asymptomatic | 03/05/2020 | neg. |
| Hajdúszoboszló (HBSZ) | HBSZ-TL-1 | ovine | pool, 2-6 years | III | asymptomatic | 03/05/2020 | neg. |
| Tárnok (TB) | TB1* | ovine | 3 weeks | I | asymptomatic | 03/16/2009 | **POS**. |
| Tárnok (TB) | TB2 | ovine | 3 weeks | I | asymptomatic | 03/16/2009 | **POS.** |
| Tárnok (TB) | TB3 | ovine | 3 weeks | I | asymptomatic | 03/16/2009 | **POS.** |
| Tárnok (TB) | TB4 | ovine | 3 weeks | I | asymptomatic | 03/16/2009 | neg. |
| Tárnok (TB) | TB5* | ovine | 3 weeks | I | asymptomatic | 03/16/2009 | **POS.** |
| Tárnok (TB) | TB6 | ovine | 3 weeks | I | asymptomatic | 03/16/2009 | **POS.** |
| Tárnok (TB) | TB7* | ovine | 3 weeks | I | asymptomatic | 03/16/2009 | **POS.** |
| Tárnok (TB) | TB8 | ovine | 3 weeks | I | asymptomatic | 03/16/2009 | **POS.** |
| Tárnok (TB) | TB9* | ovine | 3 weeks | I | asymptomatic | 04/02/2010 | **POS.** |
| Tárnok (TB) | TB10* | ovine | 3 weeks | I | asymptomatic | 04/02/2010 | **POS**. |
| Tárnok (TB) | TB11 | ovine | 3 weeks | I | asymptomatic | 04/02/2010 | **POS**. |
| Tárnok (TB) | TB12 | ovine | 3 weeks | I | asymptomatic | 04/02/2010 | neg. |
| Tárnok (TB) | TB13 | ovine | 3 weeks | I | asymptomatic | 04/02/2010 | neg. |
| Tárnok (TB) | TB14* | ovine | 3 weeks | I | asymptomatic | 04/02/2010 | **POS**. |
| Tárnok (TB) | TB15 | ovine | 3 weeks | I | asymptomatic | 04/02/2010 | neg. |
| Tárnok (TB) | TB16 | ovine | 3 weeks | I | asymptomatic | 04/02/2010 | **POS**. |
| Békéscsaba (ANI) | ANI-1 | ovine | adult | III | asymptomatic | 09/06/2009 | neg. |
| Békéscsaba (ANI) | ANI-2 | ovine | adult | III | asymptomatic | 09/06/2009 | neg. |
| Békéscsaba (ANI) | ANI-3 | ovine | adult | III | asymptomatic | 09/06/2009 | neg. |
| Békéscsaba (ANI) | ANI-4 | ovine | adult | III | asymptomatic | 09/06/2009 | neg. |
| Békéscsaba (ANI) | ANI-5 | ovine | adult | III | asymptomatic | 09/06/2009 | neg. |
| Békéscsaba (ANI) | ANI-6 | ovine | adult | III | asymptomatic | 09/06/2009 | neg. |
| Békéscsaba (ANI) | ANI-7 | ovine | adult | III | asymptomatic | 09/06/2009 | neg. |
| Békéscsaba (ANI) | ANI-8 | ovine | adult | III | asymptomatic | 09/06/2009 | neg. |
| Békéscsaba (ANI) | ANI-9 | ovine | adult | III | asymptomatic | 09/06/2009 | neg. |
| Békéscsaba (ANI) | ANI-10 | ovine | adult | III | asymptomatic | 09/06/2009 | neg. |
| Békéscsaba (ANI) | ANI-11 | ovine | adult | III | asymptomatic | 09/06/2009 | neg. |
| Békéscsaba (ANI) | ANI-12 | ovine | adult | III | asymptomatic | 09/06/2009 | neg. |
| Aranyosgadány (AGK) | AGK-1 | caprine | >12 months | III | asymptomatic | 04/23/2020 | neg. |
| Aranyosgadány (AGK) | AGK-2 | caprine | >12 months | III | asymptomatic | 04/23/2020 | neg. |
| Aranyosgadány (AGK) | AGK-3 | caprine | >12 months | III | asymptomatic | 04/23/2020 | neg. |
| Aranyosgadány (AGK) | AGK-4 | caprine | >12 months | III | asymptomatic | 04/23/2020 | neg. |
| Aranyosgadány (AGK) | AGK-5 | caprine | >12 months | III | asymptomatic | 04/23/2020 | neg. |
| Aranyosgadány (AGK) | AGK-6 | caprine | >12 months | III | asymptomatic | 04/23/2020 | neg. |
| Aranyosgadány (AGK) | AGK-7 | caprine | >12 months | III | asymptomatic | 04/23/2020 | neg. |
| Aranyosgadány (AGK) | AGK-8 | caprine | >12 months | III | asymptomatic | 04/23/2020 | neg. |
| Aranyosgadány (AGK) | AGK-9 | caprine | 2-12 months | II | asymptomatic | 04/23/2020 | neg. |
| Aranyosgadány (AGK) | AGK-10 | caprine | 2-12 months | II | asymptomatic | 04/23/2020 | neg. |
| Aranyosgadány (AGK) | AGK-11 | caprine | 2-12 months | II | asymptomatic | 04/23/2020 | neg. |
| Aranyosgadány (AGK) | AGK-12 | caprine | 2-12 months | II | asymptomatic | 04/23/2020 | neg. |
| Aranyosgadány (AGK) | AGK-13 | caprine | 2-12 months | II | diarrhoeic | 04/23/2020 | neg. |
| Aranyosgadány (AGK) | AGK-14 | caprine | 2-12 months | II | asymptomatic | 04/23/2020 | neg. |
| Aranyosgadány (AGK) | AGK-15 | caprine | 2-12 months | II | asymptomatic | 04/23/2020 | neg. |
| Aranyosgadány (AGK) | AGK-16 | caprine | 2-12 months | II | diarrhoeic | 04/23/2020 | neg. |
| Győrszentiván (KT) | KT-FI-1 | caprine | 2-4 years | III | asymptomatic | 05/11/2020 | neg. |
| Győrszentiván (KT) | KT-FI-2 | caprine | 2-4 years | III | asymptomatic | 05/11/2020 | neg. |
| Győrszentiván (KT) | KT-FI-3 | caprine | 2-4 years | III | asymptomatic | 05/11/2020 | neg. |
| Győrszentiván (KT) | KT-FI-4 | caprine | 2-4 years | III | asymptomatic | 05/11/2020 | neg. |
| Győrszentiván (KT) | KT-FI-5 | caprine | 2-4 years | III | asymptomatic | 05/11/2020 | neg. |
| Győrszentiván (KT) | KT-FII-1 | caprine | 1 years | III | asymptomatic | 05/11/2020 | neg. |
| Győrszentiván (KT) | KT-FII-2 | caprine | 1 years | III | asymptomatic | 05/11/2020 | neg. |
| Győrszentiván (KT) | KT-FII-3 | caprine | 1 years | III | asymptomatic | 05/11/2020 | neg. |
| Győrszentiván (KT) | KT-FII-4 | caprine | 1 years | III | asymptomatic | 05/11/2020 | neg. |
| Győrszentiván (KT) | KT-FII-5 | caprine | 1 years | III | asymptomatic | 05/11/2020 | neg. |
| Győrszentiván (KT) | KT-G1* | caprine | 3-4 months | II | diarrhoeic | 05/11/2020 | **POS**. |
| Győrszentiván (KT) | KT-G2 | caprine | 3-4 months | II | diarrhoeic | 05/11/2020 | **POS**. |
| Győrszentiván (KT) | KT-G3* | caprine | 3-4 months | II | diarrhoeic | 05/11/2020 | **POS**. |
| Győrszentiván (KT) | KT-G4 | caprine | 3-4 months | II | diarrhoeic | 05/11/2020 | **POS**. |
| Győrszentiván (KT) | KT-G5* | caprine | 3-4 months | II | diarrhoeic | 05/11/2020 | **POS**. |
| Győrszentiván (KT) | KT-G6 | caprine | 3-4 months | II | asymptomatic | 05/11/2020 | **POS**. |
| Győrszentiván (KT) | KT-G7 | caprine | 3-4 months | II | asymptomatic | 05/11/2020 | **POS**. |
| Győrszentiván (KT) | KT-G8 | caprine | 3-4 months | II | asymptomatic | 05/11/2020 | **POS**. |
| Győrszentiván (KT) | KT-G9 | caprine | 3-4 months | II | asymptomatic | 05/11/2020 | **POS**. |
| Győrszentiván (KT) | KT-G10 | caprine | 3-4 months | II | asymptomatic | 05/11/2020 | **POS**. |
| Győrszentiván (KT) | KT-FG-2 | caprine | 4-6 weeks | I | asymptomatic | 05/11/2020 | neg. |
| Győrszentiván (KT) | KT-FG-3 | caprine | 4-6 weeks | I | diarrhoeic | 05/11/2020 | neg. |
| Győrszentiván (KT) | KT-FG-4 | caprine | 4-6 weeks | I | asymptomatic | 05/11/2020 | neg. |
| Győrszentiván (KT) | KT-FG-5 | caprine | 2-4 weeks | I | diarrhoeic | 05/11/2020 | neg. |
| Győrszentiván (KT) | KT-FG-6 | caprine | 4-6 weeks | I | asymptomatic | 05/11/2020 | neg. |
| Győrszentiván (KT) | KT-FG-7 | caprine | 2-4 weeks | I | diarrhoeic | 05/11/2020 | neg. |
| Győrszentiván (KT) | KT-FG-8 | caprine | 4-6 weeks | I | asymptomatic | 05/11/2020 | neg. |
| Győrszentiván (KT) | KT-FG-9 | caprine | 2-4 weeks | I | diarrhoeic | 05/11/2020 | neg. |
| Győrszentiván (KT) | KT-FG-10 | caprine | 4-6 weeks | I | asymptomatic | 05/11/2020 | neg. |
| Nagyhegy (NH) | NH-1 | caprine | adult | III | asymptomatic | 05/11/2020 | neg. |
| Nagyhegy (NH) | NH-2 | caprine | adult | III | asymptomatic | 05/11/2020 | neg. |
| Nagyhegy (NH) | NH-3 | caprine | adult | III | asymptomatic | 05/11/2020 | neg. |
| Nagyhegy (NH) | NH-4 | caprine | adult | III | asymptomatic | 05/11/2020 | neg. |
| Nagyhegy (NH) | NH-5 | caprine | adult | III | asymptomatic | 05/11/2020 | neg. |
| Rudabánya (K) | K-1 | caprine | 2-12 months | II | asymptomatic | 06/25/2008 | neg. |
| Rudabánya (K) | K-2 | caprine | 2-12 months | II | asymptomatic | 06/25/2008 | neg. |
| Rudabánya (K) | K-3* | caprine | 2-12 months | II | asymptomatic | 06/25/2008 | **POS.** |
| Rudabánya (K) | K-4 | caprine | 2-12 months | II | asymptomatic | 06/25/2008 | neg. |
| Rudabánya (K) | K-5 | caprine | 2-12 months | II | asymptomatic | 06/25/2008 | neg. |
| Rudabánya (K) | K-6 | caprine | 2-12 months | II | asymptomatic | 06/25/2008 | neg. |
| Rudabánya (K) | K-7 | caprine | 2-12 months | II | asymptomatic | 06/25/2008 | neg. |
| Rudabánya (K) | K-8 | caprine | 2-12 months | II | asymptomatic | 06/25/2008 | neg. |
| Rudabánya (K) | K-9 | caprine | 2-12 months | II | asymptomatic | 06/25/2008 | neg. |
| Rudabánya (K) | K-10 | caprine | 2-12 months | II | asymptomatic | 06/25/2008 | neg. |
| Rudabánya (K) | K-11 | caprine | 2-12 months | II | asymptomatic | 06/25/2008 | neg. |
| Rudabánya (K) | K-12 | caprine | 2-12 months | II | asymptomatic | 06/25/2008 | neg. |
| Hajdúböszörmény (HB) | HB-7369 | bovine | 1-2 months | I | asymptomatic | 03/05/2020 | neg. |
| Hajdúböszörmény (HB) | HB-7660 | bovine | 1-2 months | I | asymptomatic | 03/05/2020 | neg. |
| Hajdúböszörmény (HB) | HB-7373 | bovine | 1-2 months | I | asymptomatic | 03/05/2020 | neg. |
| Hajdúböszörmény (HB) | HB-7431 | bovine | 1-2 months | I | diarrhoeic | 03/05/2020 | neg. |
| Hajdúböszörmény (HB) | HB-7396 | bovine | > 1 month | I | diarrhoeic | 03/05/2020 | neg. |
| Hajdúböszörmény (HB) | HB-7693 | bovine | 1-2 months | I | asymptomatic | 03/05/2020 | neg. |
| Hajdúböszörmény (HB) | HB-7433 | bovine | 1-2 months | I | asymptomatic | 03/05/2020 | neg. |
| Hajdúböszörmény (HB) | HB-7248 | bovine | 1-2 months | I | asymptomatic | 03/05/2020 | neg. |
| Hajdúböszörmény (HB) | HB-7615 | bovine | 1-2 months | I | diarrhoeic | 03/05/2020 | neg. |
| Hajdúböszörmény (HB) | HB-7315 | bovine | 1-2 months | I | asymptomatic | 03/05/2020 | neg. |
| Hajdúböszörmény (HB) | HB-7657 | bovine | 1-2 months | I | diarrhoeic | 03/05/2020 | neg. |
| Hajdúböszörmény (HB) | HB-7500 | bovine | 1-2 months | I | asymptomatic | 03/05/2020 | neg. |
| Hajdúböszörmény (HB) | HB-7673 | bovine | 1-2 months | I | diarrhoeic | 03/05/2020 | neg. |
| Hajdúböszörmény (HB) | HB-E-1 | bovine | adult | II | asymptomatic | 03/05/2020 | neg. |
| Hajdúböszörmény (HB) | HB-7627 | bovine | 1-2 months | I | asymptomatic | 03/05/2020 | neg. |
| Hajdúböszörmény (HB) | HB-7751 | bovine | 3 days | I | diarrhoeic | 03/05/2020 | neg. |
| Hajdúböszörmény (HB) | HB-8066 | bovine | 1-2 months | I | asymptomatic | 03/05/2020 | neg. |
| Hajdúböszörmény (HB) | HB-0303 | bovine | 2 days | I | diarrhoeic | 03/05/2020 | neg. |
| Hajdúböszörmény (HB) | HB-7663 | bovine | 1-2 months | I | asymptomatic | 03/05/2020 | neg. |
| Hajdúböszörmény (HB) | HB-7394 | bovine | 1-2 months | I | asymptomatic | 03/05/2020 | neg. |
| Hajdúböszörmény (HB) | HB-P1 | bovine | adult | III | asymptomatic | 03/05/2020 | neg. |
| Nyíregyháza (NYH) | NYH-3175 | bovine | <2 months | I | asymptomatic | 03/06/2020 | neg. |
| Nyíregyháza (NYH) | NYH-3254 | bovine | <2 months | I | asymptomatic | 03/06/2020 | neg. |
| Nyíregyháza (NYH) | NYH-3257 | bovine | <2 months | I | asymptomatic | 03/06/2020 | neg. |
| Nyíregyháza (NYH) | NYH-3259 | bovine | <2 months | I | asymptomatic | 03/06/2020 | neg. |
| Nyíregyháza (NYH) | NYH-3256 | bovine | <2 months | I | asymptomatic | 03/06/2020 | neg. |
| Nyíregyháza (NYH) | NYH-GI-1 | bovine | 10 months | II | asymptomatic | 03/06/2020 | neg. |
| Nyíregyháza (NYH) | NYH-GI-2 | bovine | 10 months | II | asymptomatic | 03/06/2020 | neg. |
| Nyíregyháza (NYH) | NYH-GII-1 | bovine | 14 months | II | asymptomatic | 03/06/2020 | neg. |
| Nyíregyháza (NYH) | NYH-GII-2 | bovine | 14 months | II | asymptomatic | 03/06/2020 | neg. |
| Nyíregyháza (NYH) | NYH-GIII-1 | bovine | 16 months | III | asymptomatic | 03/06/2020 | neg. |
| Nyíregyháza (NYH) | NYH-GIII-2 | bovine | 16 months | III | asymptomatic | 03/06/2020 | neg. |
| Derecske (DR) | DR-1 | bovine | adult | III | asymptomatic | 03/05/2020 | neg. |
| Derecske (DR) | DR-2 | bovine | adult | III | asymptomatic | 03/05/2020 | neg. |
| Derecske (DR) | DR-3 | bovine | adult | III | asymptomatic | 03/05/2020 | neg. |
| Derecske (DR) | DR-4 | bovine | adult | III | asymptomatic | 03/05/2020 | neg. |
| Derecske (DR) | DR-5 | bovine | adult | III | asymptomatic | 03/05/2020 | neg. |
| Derecske (DR) | DR-6 | bovine | adult | III | asymptomatic | 03/05/2020 | neg. |
| Derecske (DR) | DR-7 | bovine | adult | III | asymptomatic | 03/05/2020 | neg. |
| Derecske (DR) | DR-8 | bovine | adult | III | asymptomatic | 03/05/2020 | neg. |
| Derecske (DR) | DR-9 | bovine | adult | III | asymptomatic | 03/05/2020 | neg. |
| Derecske (DR) | DR-10 | bovine | adult | III | asymptomatic | 03/05/2020 | neg. |
| Derecske (DR) | DR-11 | bovine | adult | III | asymptomatic | 03/05/2020 | neg. |
| Derecske (DR) | DR-B-1 | bovine | 1-2 months | I | asymptomatic | 03/05/2020 | neg. |
| Derecske (DR) | DR-B-2 | bovine | 1-2 months | I | asymptomatic | 03/05/2020 | neg. |
| Derecske (DR) | DR-B-3 | bovine | 1-2 months | I | asymptomatic | 03/05/2020 | neg. |
| Derecske (DR) | DR-B-4 | bovine | 1-2 months | I | asymptomatic | 03/05/2020 | neg. |
| Tiszavasvári (TiV) | TiV-001 | bovine | adult | III | asymptomatic | 03/05/2020 | neg. |
| Tiszavasvári (TiV) | TiV-002 | bovine | adult | III | asymptomatic | 03/05/2020 | neg. |
| Tiszavasvári (TiV) | TiV-003 | bovine | adult | III | asymptomatic | 03/05/2020 | neg. |
| Tiszavasvári (TiV) | TiV-004 | bovine | adult | III | asymptomatic | 03/05/2020 | neg. |
| Tiszavasvári (TiV) | TiV-005 | bovine | adult | III | asymptomatic | 03/05/2020 | neg. |
| Tiszavasvári (TiV) | TiV-GI-1 | bovine | 5 months | II | asymptomatic | 03/05/2020 | neg. |
| Tiszavasvári (TiV) | TiV-GI-2 | bovine | 5 months | II | asymptomatic | 03/05/2020 | neg. |
| Tiszavasvári (TiV) | TiV-GI-3 | bovine | 5 months | II | asymptomatic | 03/05/2020 | neg. |
| Tiszavasvári (TiV) | TiV-GII-1 | bovine | 3 months | II | asymptomatic | 03/05/2020 | neg. |
| Tiszavasvári (TiV) | TiV-GII-2 | bovine | 3 months | II | asymptomatic | 03/05/2020 | neg. |
| Tiszavasvári (TiV) | TiV-GIII-1 | bovine | 4 months | II | asymptomatic | 03/05/2020 | neg. |
| Tiszavasvári (TiV) | TiV-GIII-2 | bovine | 4 months | II | asymptomatic | 03/05/2020 | neg. |
| Tiszavasvári (TiV) | TiV-F1 | bovine | adult | III | asymptomatic | 03/05/2020 | neg. |
| Tiszavasvári (TiV) | TiV-007 | bovine | 1-2 months | III | asymptomatic | 03/05/2020 | neg. |
| Tiszavasvári (TiV) | TiV-6379 | bovine | 1-2 months | I | asymptomatic | 03/05/2020 | neg. |
| Tiszavasvári (TiV) | TiV-0577 | bovine | 1-2 months | I | asymptomatic | 03/05/2020 | neg. |
| Bonyhád (BH) | BH-0001 | bovine | 1-2 months | I | asymptomatic | 11/04/2019 | neg. |
| Bonyhád (BH) | BH-9812 | bovine | 1-2 months | I | asymptomatic | 11/04/2019 | neg. |
| Bonyhád (BH) | BH-9852 | bovine | 1-2 months | I | asymptomatic | 11/04/2019 | neg. |
| Bonyhád (BH) | BH-9854 | bovine | 1-2 months | I | asymptomatic | 11/04/2019 | neg. |
| Bonyhád (BH) | BH-9858 | bovine | 1-2 months | I | asymptomatic | 11/04/2019 | neg. |
| Bonyhád (BH) | BH-9861 | bovine | 1-2 months | I | asymptomatic | 11/04/2019 | neg. |
| Bonyhád (BH) | BH-9865 | bovine | 1-2 months | I | asymptomatic | 11/04/2019 | neg. |
| Bonyhád (BH) | BH-9878 | bovine | 1-2 months | I | asymptomatic | 11/04/2019 | neg. |
| Bonyhád (BH) | BH-9901 | bovine | 1-2 months | I | asymptomatic | 11/04/2019 | neg. |
| Bonyhád (BH) | BH-9955 | bovine | 1-2 months | I | asymptomatic | 11/04/2019 | neg. |
| Bonyhád (BH) | BH-9963 | bovine | 1-2 months | I | asymptomatic | 11/04/2019 | neg. |
| Bonyhád (BH) | BH-9971 | bovine | 1-2 months | I | asymptomatic | 11/04/2019 | neg. |
| Bonyhád (BH) | BH-9974 | bovine | 1-2 months | I | asymptomatic | 11/04/2019 | neg. |
| Bonyhád (BH) | BH-9989 | bovine | 1-2 months | I | asymptomatic | 11/04/2019 | neg. |
| Bonyhád (BH) | BH-9993 | bovine | 2-12 months | II | asymptomatic | 11/04/2019 | neg. |
| Bonyhád (BH) | BH-9994 | bovine | 2-12 months | II | asymptomatic | 11/04/2019 | neg. |
| Tevel (TV) | TV-9682 | bovine | 1-2 months | I | asymptomatic | 11/04/2019 | neg. |
| Tevel (TV) | TV-9686 | bovine | 1-2 months | I | asymptomatic | 11/04/2019 | neg. |
| Tevel (TV) | TV-9703 | bovine | 1-2 months | I | asymptomatic | 11/04/2019 | neg. |
| Tevel (TV) | TV-9728 | bovine | 1-2 months | I | asymptomatic | 11/04/2019 | neg. |
| Tevel (TV) | TV-9733 | bovine | 1-2 months | I | asymptomatic | 11/04/2019 | neg. |
| Tevel (TV) | TV-9736 | bovine | 1-2 months | I | asymptomatic | 11/04/2019 | neg. |
| Tevel (TV) | TV-9738 | bovine | 1-2 months | I | asymptomatic | 11/04/2019 | neg. |
| Tevel (TV) | TV-9743 | bovine | 1-2 months | I | asymptomatic | 11/04/2019 | neg. |
| Tevel (TV) | TV-9746 | bovine | 1-2 months | I | asymptomatic | 11/04/2019 | neg. |
| Tevel (TV) | TV-9760 | bovine | 1-2 months | I | asymptomatic | 11/04/2019 | neg. |
| Tevel (TV) | TV-9764 | bovine | 1-2 months | I | asymptomatic | 11/04/2019 | neg. |
| Tevel (TV) | TV-9772 | bovine | 1-2 months | I | asymptomatic | 11/04/2019 | neg. |
| Tevel (TV) | TV-9774 | bovine | 1-2 months | I | asymptomatic | 11/04/2019 | neg. |
| Tevel (TV) | TV-9779 | bovine | 1-2 months | I | asymptomatic | 11/04/2019 | neg. |
| Tevel (TV) | TV-9785 | bovine | 2-12 months | II | asymptomatic | 11/04/2019 | neg. |
| Tevel (TV) | TV-9787 | bovine | 2-12 months | II | asymptomatic | 11/04/2019 | neg. |
| Tevel (TV) | TV-9789 | bovine | 2-12 months | II | asymptomatic | 11/04/2019 | neg. |
| Egyházasfalu (EF) | EF-1 | swine | 3 months | II | asymptomatic | 08/17/2016 | neg. |
| Egyházasfalu (EF) | EF-2 | swine | 3 months | II | asymptomatic | 08/17/2016 | neg. |
| Egyházasfalu (EF) | EF-3 | swine | 3 months | II | asymptomatic | 08/17/2016 | neg. |
| Egyházasfalu (EF) | EF-4 | swine | 3 months | II | asymptomatic | 08/17/2016 | neg. |
| Egyházasfalu (EF) | EF-5 | swine | 3 months | II | asymptomatic | 08/17/2016 | neg. |
| Egyházasfalu (EF) | EF-6 | swine | 3 months | II | asymptomatic | 08/17/2016 | neg. |
| Egyházasfalu (EF) | EF-7 | swine | 3 months | II | asymptomatic | 08/17/2016 | neg. |
| Egyházasfalu (EF) | EF-8 | swine | 3 months | II | asymptomatic | 08/17/2016 | neg. |
| Egyházasfalu (EF) | EF-9 | swine | 3 months | II | asymptomatic | 08/17/2016 | neg. |
| Egyházasfalu (EF) | EF-10 | swine | 3 months | II | asymptomatic | 08/17/2016 | neg. |
| Tázlár ('TM) | TM-1121 | swine | 7-21 days | I | paraplegic | 01/2013 | neg. |
| Tázlár ('TM) | TM-1130 | swine | 7-21 days | I | paraplegic | 01/2013 | neg. |
| Tázlár ('TM) | TM-1205 | swine | 7-21 days | I | paraplegic | 01/2013 | neg. |
| Nagyszokoly (NSzK) | NSzK1 | swine | 11-12 months | II | paraplegic | 01/23/2013 | neg. |
| Kevermes (KEV) | KEV-1 | swine | 4-5 months | II | paraplegic | 09/05/2013 | neg. |
| Kevermes (KEV) | KEV-2 | swine | 4-5 months | II | paraplegic | 09/05/2013 | neg. |
| Kevermes (KEV) | KEV-3 | swine | 4-5 months | II | paraplegic | 09/05/2013 | neg. |
| Zsana | Zs-1 | swine | 2-3 months | II | paraplegic | 04/14/2013 | neg. |
| Zsana | Zs-2 | swine | 2-3 months | II | paraplegic | 04/14/2013 | neg. |
| Zsana | Zs-3 | swine | 2-3 months | II | paraplegic | 04/14/2013 | neg. |
| Balmazújváros (BUV) | BUV-1 | swine | 1 month | I | paraplegic | 04/24/2013 | neg. |
| Balmazújváros (BUV) | BUV-2 | swine | 1 month | I | paraplegic | 04/24/2013 | neg. |
| Székelyszabar (SZ) | SZ1K | swine | >12 months | III | asymptomatic | 01/2013 | neg. |
| Székelyszabar (SZ) | SZ2K | swine | >12 months | III | asymptomatic | 01/2013 | neg. |
| Székelyszabar (SZ) | SZK3 | swine | >12 months | III | asymptomatic | 01/2013 | neg. |
| Székelyszabar (SZ) | SZK4 | swine | >12 months | III | asymptomatic | 01/2013 | neg. |
| Székelyszabar (SZ) | SZ1M | swine | 7-21 days | I | paraplegic | 01/2013 | neg. |
| Székelyszabar (SZ) | SZ2M | swine | 7-21 days | I | paraplegic | 01/2013 | neg. |
| Székelyszabar (SZ) | SZ3M | swine | 7-21 days | I | paraplegic | 01/2013 | neg. |
| Székelyszabar (SZ) | SZ4M | swine | 7-21 days | I | paraplegic | 01/2013 | neg. |
